# Genetic prevention of lymphoma in p53 knockout mice allows the early development of p53-related sarcomas

**DOI:** 10.18632/oncotarget.2650

**Published:** 2014-11-18

**Authors:** Lorena Landuzzi, Marianna L. Ianzano, Giordano Nicoletti, Arianna Palladini, Valentina Grosso, Dario Ranieri, Massimiliano Dall'Ora, Elena Raschi, Roberta Laranga, Marco Gambarotti, Piero Picci, Carla De Giovanni, Patrizia Nanni, Pier-Luigi Lollini

**Affiliations:** ^1^ Laboratory of Experimental Oncology, Rizzoli Orthopedic Institute, Bologna, Italy; ^2^ PROMETEO Laboratory, STB, RIT Department, Rizzoli Orthopedic Institute, Bologna, Italy; ^3^ Laboratory of Immunology and Biology of Metastasis, Department of Experimental, Diagnostic and Specialty Medicine, University of Bologna, Bologna Italy; ^4^ Anatomy and Pathological Histology, Rizzoli Orthopedic Institute, Bologna, Italy

**Keywords:** p53-KO mice, Rag2KO/Il2rgKO mice, lymphoma, hemangiosarcoma, osteosarcoma

## Abstract

Homozygous knockout of p53 in mice leads to early mortality from lymphoma, with almost complete penetrance, thus hampering studies of other tumor histotypes related to p53 alterations. To avoid lymphoma development, we crossed p53 knockout mice (BALB-p53 mice) with alymphocytic BALB/c Rag2^−/−^;Il2rg^−/−^ (RGKO) mice. We compared the tumor spectrum of homozygous (BALB-p53^−/−^) and heterozygous (BALB-p53^+/−^) mice with alymphocytic mice (RGKO-p53^−/−^ and RGKO-p53^+/−^). Lymphoma incidence in BALB-p53^−/−^ mice exceeded 80%, whereas in RGKO-p53^−/−^ it was strongly reduced. The prevalent tumor of RGKO-p53^−/−^ mice was hemangiosarcoma (incidence over 65% in both sexes, mean latency 18 weeks), other tumors included soft tissue sarcomas (incidence ~10%), lung and mammary carcinomas. Tumor spectrum changes occurred also in p53 heterozygotes, in which lymphomas are relatively rare (~20%). RGKO-p53^+/−^ had an increased incidence of hemangiosarcomas, reaching ~30%, and females had an increased incidence of osteosarcomas, reaching ~20%. Osteosarcomas shared with the corresponding human tumors the involvement of limbs and a high metastatic ability, mainly to the lungs. Specific alterations in the expression of p53-related genes (p16Ink4a, p19Arf, p15Ink4b, p21Cip1) were observed. Genetic prevention of lymphoma in p53 knockout mice led to new models of sarcoma development, available for studies on hemangiosarcoma and osteosarcoma onset and metastatization.

## INTRODUCTION

The expression of the tumor suppressor gene p53 is altered by mutation and other mechanisms in a large proportion and in a wide variety of human cancers [1, 2].

p53 knockout mice are a precious tool to investigate the mechanisms of carcinogenesis in the absence of p53. However, p53 knockout mice are prone to the early development of lymphomas, in particular of thymic origin [3–5], hence the study of other solid tumors is greatly hampered in these mice.

This is a general problem in mice harboring cancer syndromes. Early onset and lethality of a given tumor type can preempt the study of other tumors arising later in life. It is a problem mostly unsolvable, even though empirical solutions can be found in some instances by changing the genetic background, or through direct or indirect modifications of the gene of interest, for example using tissue-specific Cre-lox systems [4].

In the case of p53, variations in tumor spectrum were obtained either using mutant p53 genes with gain of function properties [6–9] or through conditional inactivation of p53 in specific tissues [4, 10–14]. Gain of function mutants are certainly informative for what concerns the corresponding human mutations, but not for those conditions, such as the Li-Fraumeni syndrome, in which biallelic losses are frequent lesions [4]. On the other hand, inactivation of p53 in prespecified tissues leaves open the question of which tumors could result from an organism-wide lack of p53.

For what concerns the predominant lymphoma of p53 knockout mice, the cell population at risk of neoplastic transformation, i.e. lymphocytes, is not indispensable for life, at least under sterile conditions, and various alymphocytic mouse lines were obtained through genetic manipulation. Therefore, we reasoned that cross-breeding of p53 knockout mice with alymphocytic mice could avoid lymphoma development. We show here that p53 knockout mice crossed with Rag2^−/−^;Il2rg^−/−^ mice (RGKO-p53^−/−^ mice), which lack T, B and NK cells [15], had a strong impairment of lymphoma development, thus allowing the study of solid tumors, mainly sarcomas, caused by the lack of p53.

## RESULTS

### Tumor incidence and survival of alymphocytic p53 knockout mice

BALB-p53^+/–^ mice were crossed with Rag2^−/−^;Il2rg^−/−^ (RGKO) mice. Knockout of Rag2 (recombinase activating gene 2) prevents V(D)J recombination, and knockout of Il2rg (common gamma chain of IL-2, IL-4, IL-7, IL-9, IL-15 and IL-21 receptors) blocks signal transduction by cytokine receptors. RGKO mice display a complete absence of mature T, B and NK cells [15].

**Figure 1:**
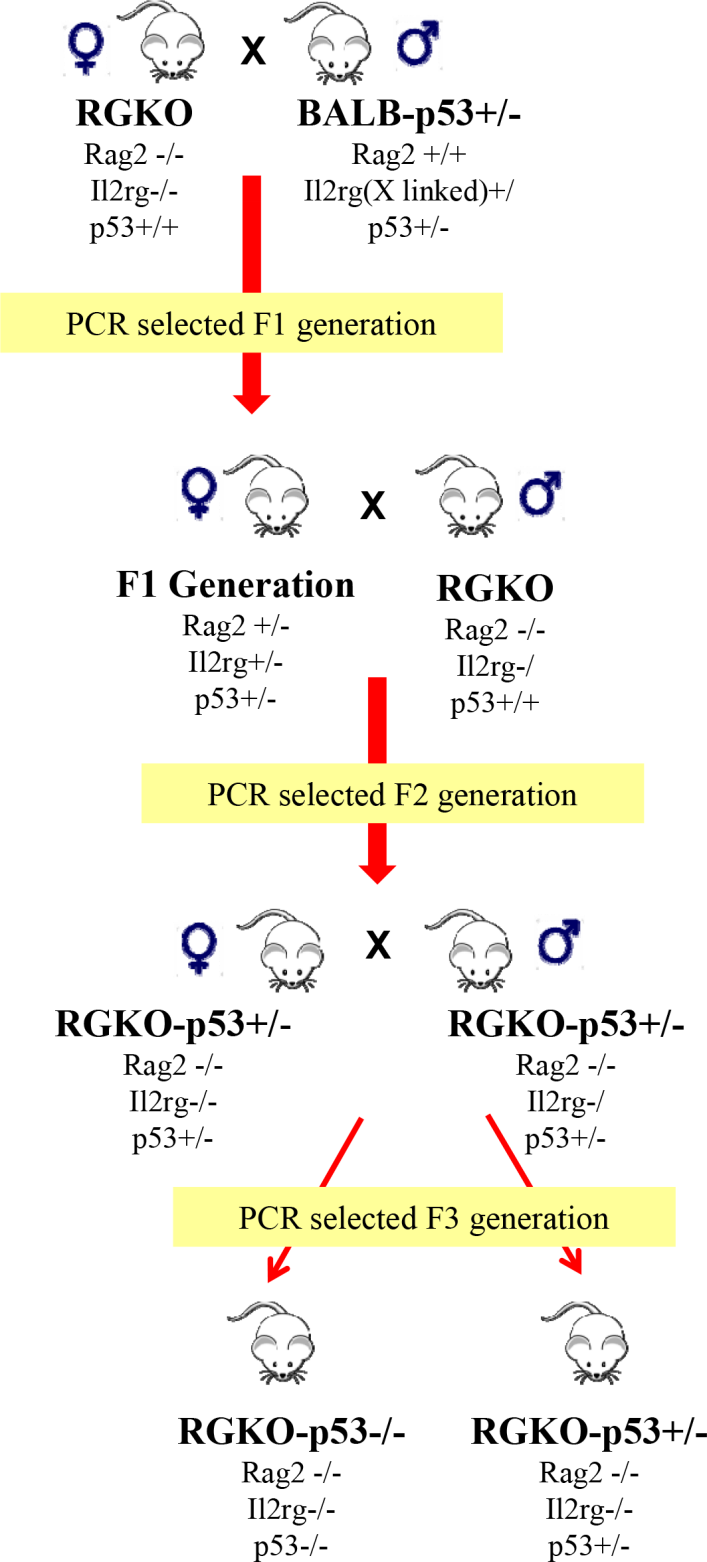
Obtainment of alymphocytic p53 knockout mice Male BALB-p53^+/−^ mice were crossed with female Rag2^−/−^;Il2rg^−/−^ (RGKO) mice to obtain the F1 generation, the progeny was genotyped by PCR. Taking advantage of the X-linked Il2rg gene, female F1 mice carrying the genotype indicated in figure were backcrossed with male RGKO mice. At the F2 generation PCR genotyping allowed to select RGKO-p53^+/−^ male and female mice that were backcrossed to obtain, from the F3 generation, female and male RGKO mice with homozygous or heterozygous p53 ablation, as determined by PCR genotyping.

Rag2^+/−^,Il2rg^+/−^,p53^+/−^ PCR-selected female mice of the F1 generation were backcrossed with male RGKO mice to obtain and select the F2 generation with homozygous RGKO and p53^+/−^ genotype (Fig. 1). F3 generation was obtained by crossing F2 RGKO-p53^+/−^ male and female mice. As expected, a quarter of the progeny had a RGKO-p53^−/−^ genotype.

**Figure 2:**
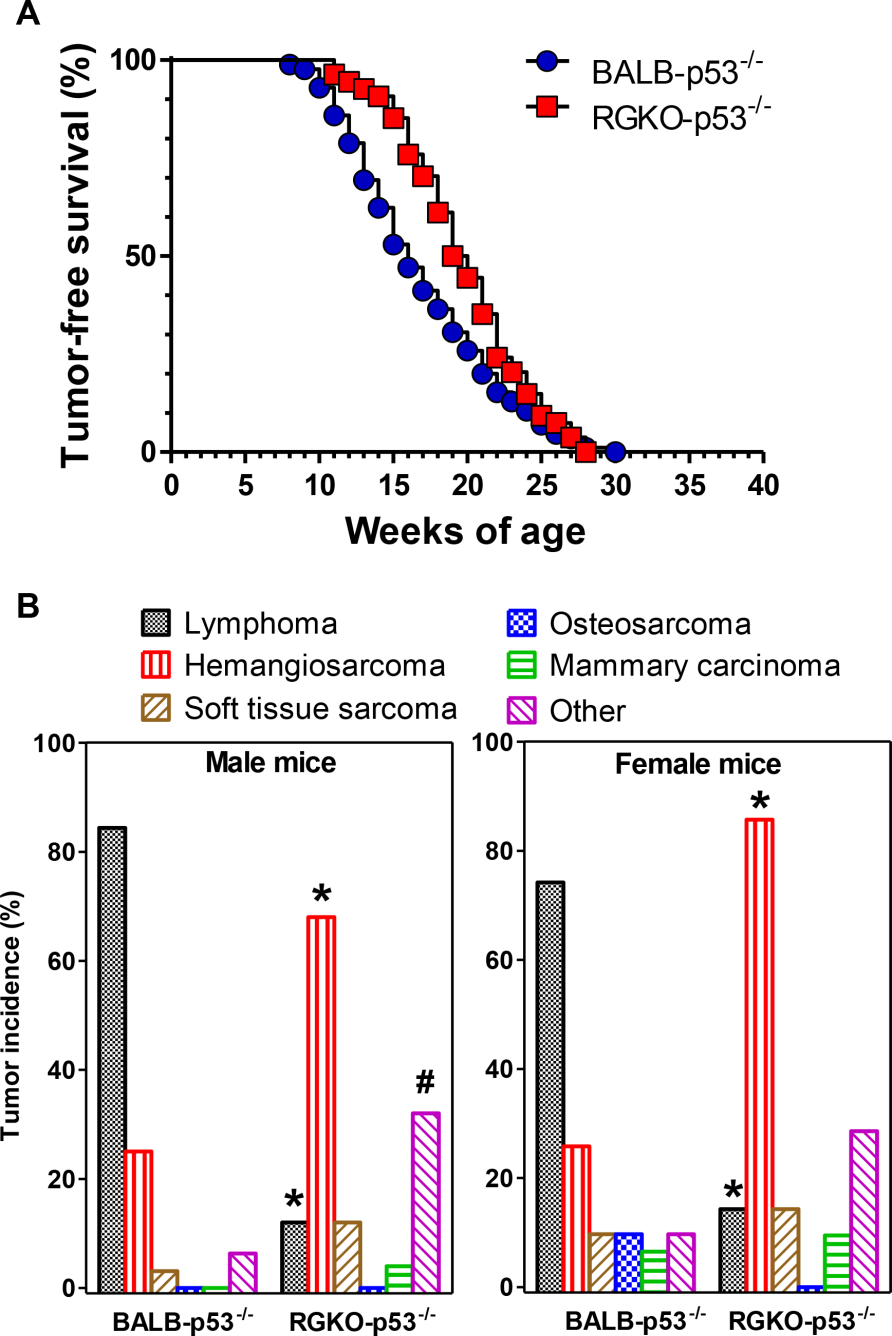
Tumor-free survival and tumor histotype incidence in homozygous p53 knockout mice **(A)** Kaplan-Meier tumor-free survival curves of BALB-p53^−/−^ (85 mice) and RGKO-p53^−/−^ mice (54 mice) were significantly different by the logrank Mantel-Haenszel test, *p* < 0.01. **(B)** Tumor histotype frequencies in BALB-p53^−/−^ and RGKO-p53^−/−^ mice. Alymphocytic RGKO-p53^−/−^ mice showed significantly reduced lymphoma incidence and significantly increased hemangiosarcoma onset compared to BALB-p53^−/−^ mice (* = *p* < 0.01, χ^2^ analysis; male BALB n = 32, male RGKO n = 25; female BALB n = 31, female RGKO n = 21). The category “Other” included tumors of the lungs, genitourinary tract, salivary glands, kidneys and liver, and its incidence in males was significantly higher in RGKO-p53^−/−^ compared to BALB-p53^−/−^ mice (# = *p* < 0.05, χ^2^ analysis). In RGKO-p53^−/−^ mice global incidence of carcinomas was significantly lower than that of sarcomas (34% *vs*. 82%, respectively, *p* < 0.01 by χ^2^ analysis). Mice showing multiple primary tumors were counted in each tumor type.

All p53^−/−^ mice developed progressive tumors by 30 weeks of age (Fig. 2A). Tumor onset was slightly, but significantly, delayed in RGKO-p53 mice: BALB-p53^−/−^ mice had a median tumor-free survival time of 16 weeks, while RGKO-p53^−/−^ mice had a median of 19.5 weeks, however, after 20 weeks of age, the tumor-free survival curves overlapped.

### Prevention of lymphoma development

More then three quarters of BALB-p53^−/−^ mice developed lymphomas, mostly of thymic origin. A minority of mice developed sarcomas or carcinomas (Fig. 2B), frequently in combination with lymphoid tumors.

The incidence of lymphomas in RGKO-p53^−/−^ mice was strongly reduced and became a sporadic event, possibly resulting from the neoplastic transformation of immature precursors (see below).

**Figure 3:**
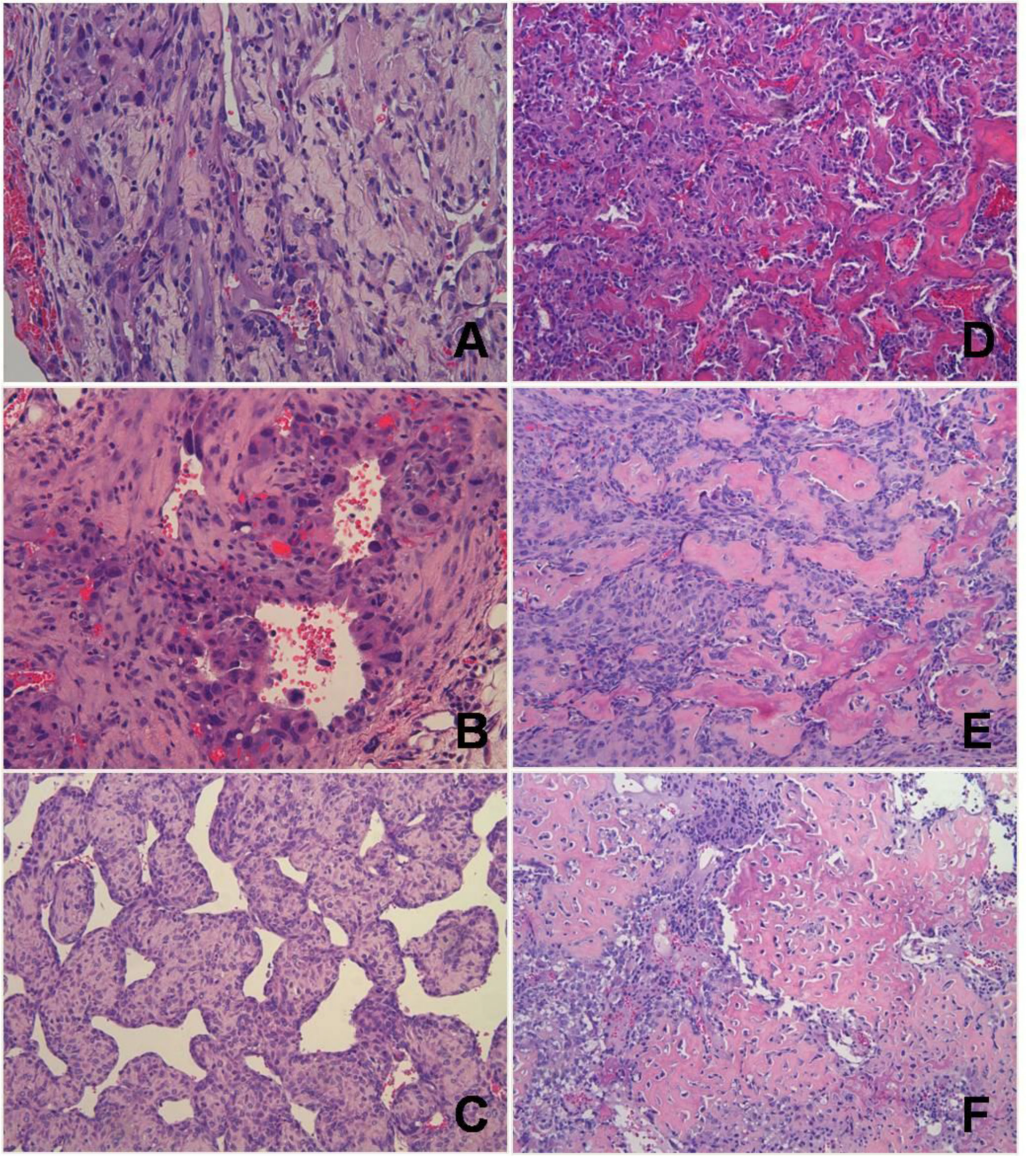
Representative histology of vascular and perivascular tumors of RGKO-p53^−/−^ mice and of osteosarcomas of RGKO-p53^+/−^ mice **(A)** Soft tissue lesion of the genito-urinary tract showing a tumor of vascular origin with features of an angiosarcoma; mHS-1 cell line was derived from this tumor. **(B)** Soft tissue lesion of the cervico-nuchal region with malignant cells surrounding vessels that suggest a diagnosis of angiosarcoma; mHS-2 cell line was established from this tumor. **(C)** Soft tissue lesion of the right forelimb showing an histological pattern of hemangiopericytoma/solitary fibrous tumor. **(D)** Ossified mass of the right hind limb, osteosarcoma grade 3. The osteosarcoma cell line mOS-1 was derived from this tumor. **(E)** Ossified mass of the left hind limb, osteosarcoma grade 4. The osteosarcoma cell line mOS-2 was derived from this tumor. **(F)** Ossified mass of the right hind limb, osteosarcoma grade 4, showing presence of lau-like cells. All pictures: hematoxylin and eosin staining, magnification ×200.

**Figure 4:**
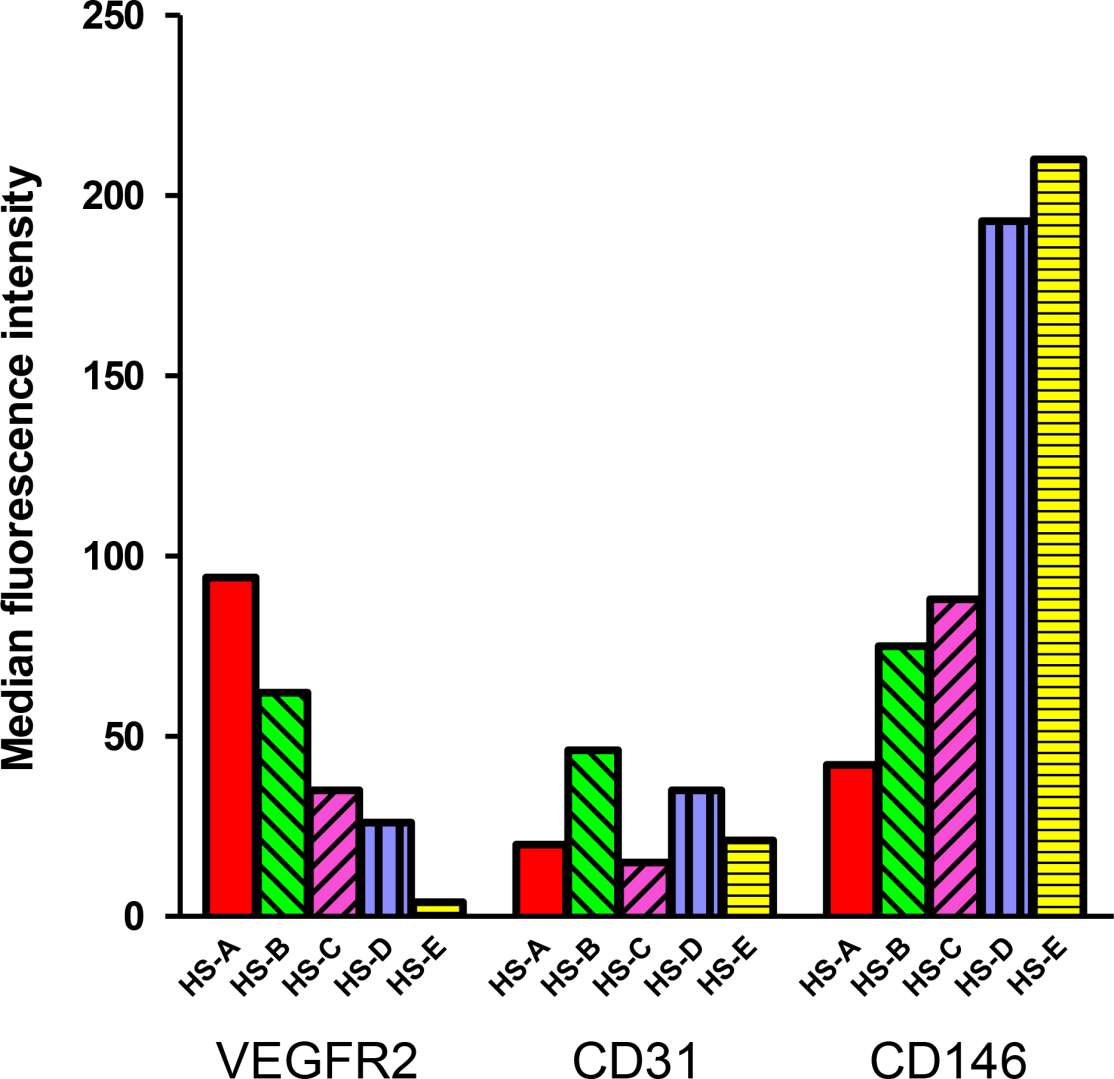
Expression of endothelial markers in hemangiosarcomas Expression of VEGF-R2, CD31 and CD146 in five primary hemangiosarcomas determined by immunofluorescence and cytofluorometric analysis, after mechanical and enzymatical dissociation of the tumor. Bars represent the median fluorescence intensity of each marker in each different hemangiosarcoma.

**Figure 5:**
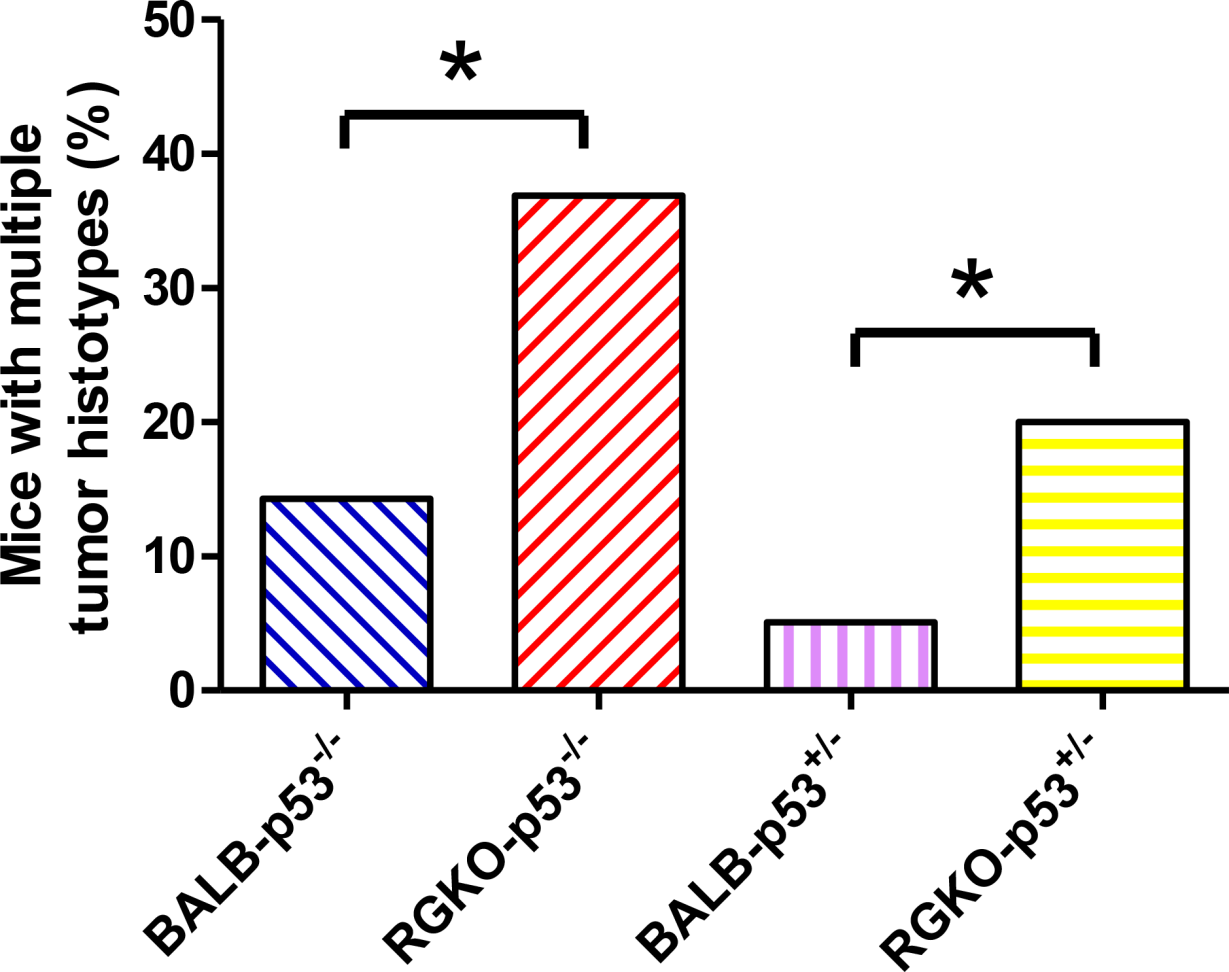
Mice bearing multiple tumor histotypes The proportion of mice bearing multiple tumor histotypes was significantly higher in RGKO-p53 compared to BALB-p53 (*p < 0.05, χ^2^ analysis).

Prevention of lymphoma allowed the onset of a variety of other tumor histotypes, mostly sarcomas (Fig 2B). Over two-thirds of both female and male RGKO-p53^−/−^ mice developed spontaneous malignant vascular tumors, here collectively indicated as hemangiosarcomas, in skin, muscles, liver, lungs, peritoneal membranes, intestine, spleen and genito-urinary tract. Most vascular tumors of RGKO-p53^−/−^ mice showed the features of aggressive angiosarcomas (Fig. 3 A-C) expressing endothelial markers, such as VEGF-R2, CD31 and CD146, as determined by FACS analysis (Fig. 4). About half of mice carrying hemangiosarcomas had a multifocal or disseminated disease. The general tumor burden (number of tumor sites) of BALB-p53^−/−^ and RGKO-p53^−/−^ mice were similar (median 2, range 1–8, and median 2, range 1–11, respectively). However the proportion of mice with multiple tumor histotypes was significantly increased in RGKO-p53^−/−^ mice (Fig. 5).

**Figure 6:**
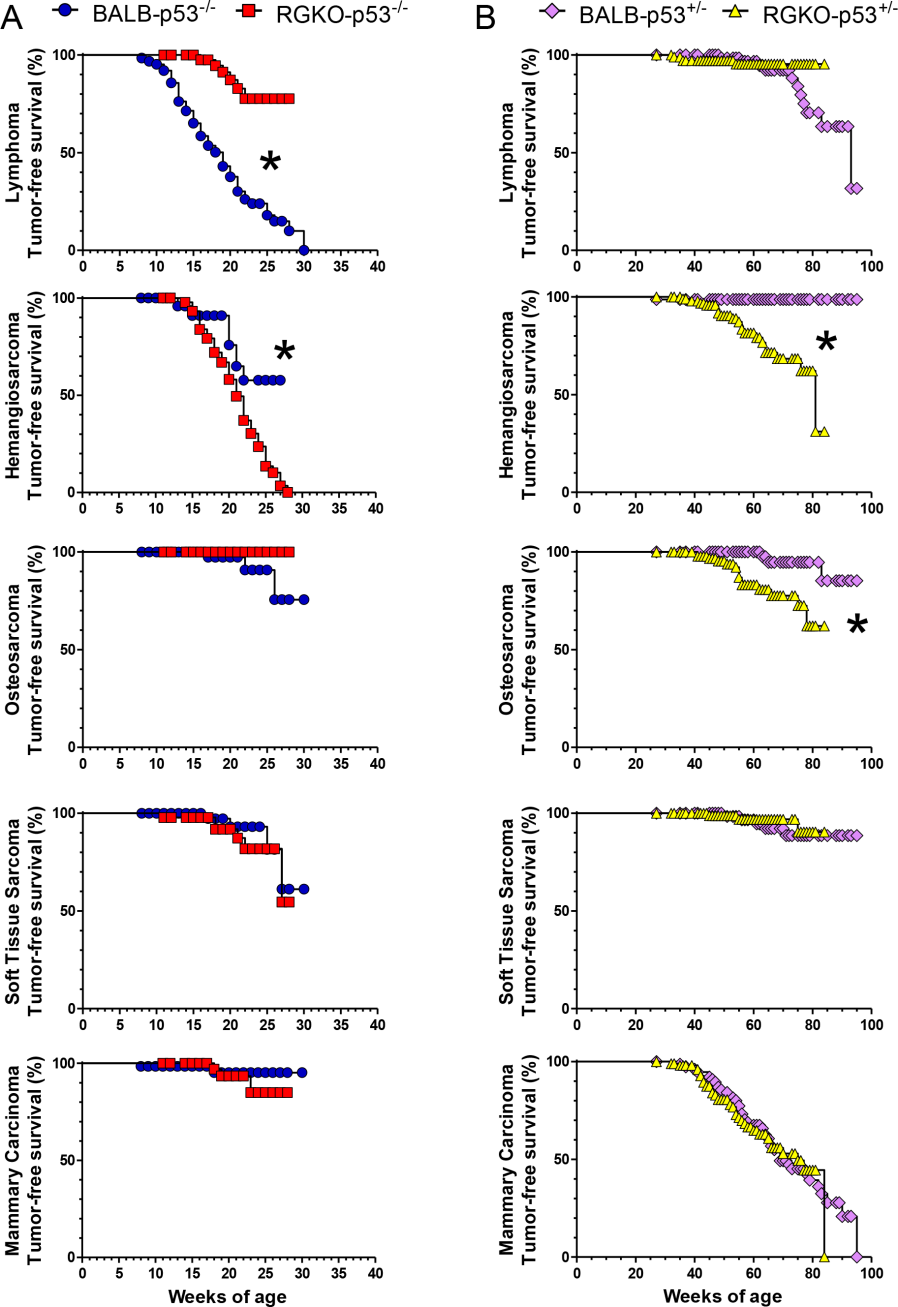
Tumor-free survival rate by individual tumor histotype of homozygous and heterozygous p53 knockout mice Kaplan-Meier tumor-free survival curves of p53^−/−^ **(A)** and p53^+/−^ **(B)** mice. Stars denote significant (*p* < 0.01) differences between BALB-p53 and RGKO-p53 mice by the logrank Mantel-Haenszel test.

Tumor-free survival curves of individual tumor histotypes showed that lymphoma-free survival and hemangiosarcoma-free survival differed significantly between BALB-p53^−/−^ and RGKO-p53^−/−^, the former developing early lymphomas and only few hemangio-sarcomas and the latter showing the opposite (Fig. 6A). Tumor-free survival curves for osteosarcoma, soft tissue sarcoma and mammary carcinoma did not differ significantly between the two strains.

Interestingly, in RGKO-p53^−/−^ mice, the global incidence of carcinomas (34%) was significantly lower than that of sarcomas (82%), thus suggesting that neoplastic transformation of epithelial cells was slower than that of mesenchymal cells, or required additional somatic events.

Finally we addressed the question of the origin of lymphomas in RGKO-p53^−/−^ mice. It must be kept in mind that these “alymphocytic” mice actually lack lymphocytes with a rearranged antigen receptor, but bone marrow lymphopoiesis and migration proceed normally. The rare lymphomas of RGKO-p53^−/−^ mice arose predominantly in the thymus. One case studied by flow cytometry consisted of blast cells with large forward and side scatter and an immature single-positive (ISP)-like phenotype (CD45R/B220^dim^, CD3^−^, CD4^−^, CD8^+^) [16–19]. The residual incidence of lymphomas in RGKO-p53^−/−^ mice suggests that lymphomas of p53^−/−^ mice should be re-examined to assess the role of p53 before and after rearrangement.

### Tumors of p53 heterozygous mice

**Figure 7:**
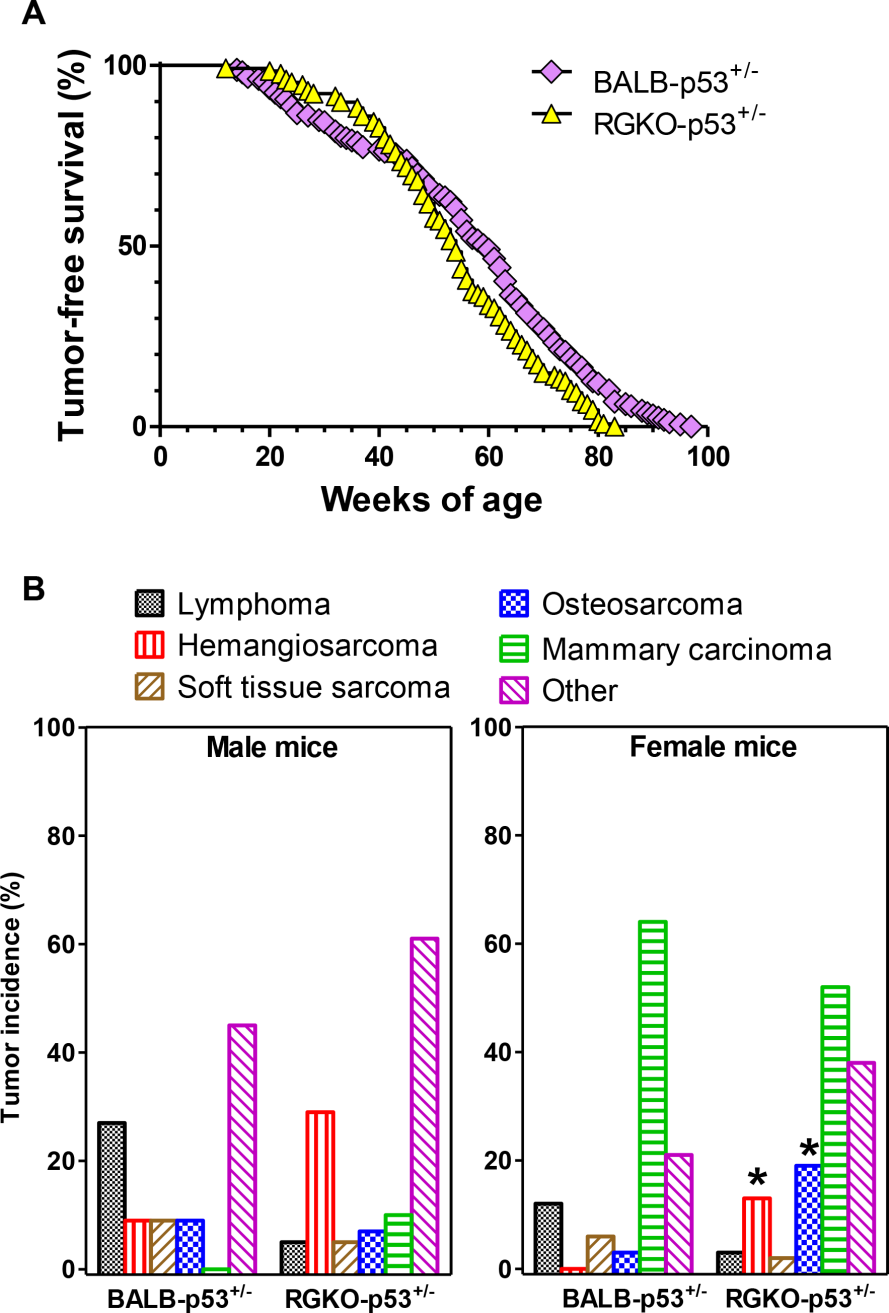
Tumor-free survival rate and tumor histotype incidence in heterozygous p53 mice **(A)** Kaplan-Meier tumor-free survival curves of BALB-p53^+/−^ (159 mice) and RGKO-p53^+/−^ mice (124 mice). Curves were significantly different by the logrank Mantel-Haenszel test, *p* < 0.01. **(B)** Tumor histotype frequencies in BALB-p53^+/−^ and RGKO-p53^+/−^ mice. Global incidence of lymphomas was significantly lower in RGKO-p53^+/−^ than in BALB-p53^+/−^ (*p* < 0.05, χ^2^ analysis male BALB n = 11, male RGKO n = 41; female BALB n = 67, female RGKO n = 64). Hemangiosarcoma incidence was significantly higher in female RGKO-p53^+/−^ compared to BALB-p53^+/−^ (*p < 0.01, χ^2^ analysis), a similar trend was observed in males, and the cumulative incidence of hemangiosarcoma was significantly increased in RGKO-p53^+/−^ mice compared to BALB-p53^+/−^ mice (*p* < 0.01, χ^2^ analysis). Osteosarcoma incidence was significantly increased only in female RGKOp53^+/−^mice, *p < 0.01 by χ^2^ analysis. The category “Other” included tumors of the genitourinary tract, adrenals, lungs, kidneys, salivary glands and liver, and its cumulative incidence in males and females was significantly higher in RGKO-p53^+/−^ than in BALB-p53^+/−^ mice (*p* < 0.01, χ^2^ analysis). Mice showing multiple primary tumors were counted in each tumor type.

The breeding scheme adopted to obtain RGKO-p53^−/−^ mice gave us the opportunity to study also p53^+/−^ heterozygotes, in which tumor development was generally much slower than in homozygotes (compare Fig. 2A and 7A). As in homozygotes, also in p53 heterozygous mice the general tumor load was similar in BALB-p53^+/−^ and RGKO-p53^+/−^(median 2, range 1–6, and median 2, range 1–10, respectively) but the proportion of mice with multiple tumor histotypes was significantly increased in RGKO-p53^+/−^ mice (Fig. 5).

Lymphomas were found in about 20% of BALB-p53^+/−^ mice, and again cross-breeding with RGKO mice prevented their onset, but the tumor spectrum of RGKO-p53^+/−^ mice showed only minor modifications (Fig. 7B). As in homozygotes, the incidence of hemangiosarcomas increased in RGKO-p53^+/−^ mice in comparison to BALB-p53^+/−^ mice of both sexes. Female RGKO-p53^+/−^ showed a distinctive increase, up to 20%, in the incidence of osteosarcoma, which became the third most common tumor in these mice (Fig. 7B). Osteosarcomas (Fig. 3 D-F) mainly affected the limbs (90% of cases) and gave rise to spontaneous metastatic dissemination, in particular to the lungs (25% of cases).

Tumor-free survival rates of BALB-p53^+/−^ and RGKO-p53^+/−^ were similar, however tumor onset was slightly but significantly faster in immunodeficient RGKO-p53^+/−^ mice than in BALB-p53^+/−^ mice (Fig. 7A). Tumor-free survival curves of individual tumor histotypes showed that hemangiosarcoma-free survival and osteosarcoma-free survival were significantly different between BALB-p53^+/−^ and RGKO-p53^+/−^mice, whereas lymphoma-free survival did not differ significantly and soft tissue sarcoma-free and mammary carcinoma-free survival almost overlapped, possibly suggesting a (weak) immunological control in long-term tumor development mainly regarding sarcomas (Fig. 6B).

### Hemangiosarcoma and osteosarcoma cell lines

To further investigate the nature of tumors arising in RGKO-p53^−/−^ mice, we derived in vitro cultures and established stable cell lines from hemangiosarcomas and osteosarcomas. Hemangiosarcoma cell lines, which in vitro expressed typical endothelial markers, such as VEGF-R2, CD31 and CD146, were poorly tumorigenic, giving rise to local tumors only after very long latency times (Tab. 1). In contrast, osteosarcoma cell lines rapidly gave rise to ossified local tumors (Tab. 1). In both cases, growth in immunocompetent mice was less efficient and slower than in immunodeficient mice, thus indicating immune recognition of cells deriving from tumors arisen in immunodeficient hosts. Distant metastases to the lungs were found in a sizeable proportion (>40%) of mice bearing local tumors, in particular in immunodeficient mice. Intravenous injections of tumor cells confirmed the faster growth and the higher malignancy of osteosarcoma cells (Tab. 2).

**Table 1:** Tumorigenic ability of hemangiosarcoma and osteosarcoma cell lines in immunocompetent BALB/c and in immunodeficient RGKO mice

**Cell line**^#^	**Host**	**Tumor**		
		**Incidence**	**Latency (Median weeks)**	Median weeks at sacrifice^*^
**mHS-3**	BALB/c	2/4 (50%)	29	58
	RGKO	5/5 (100%)	37	46
**mOS-1**	BALB/c	3/5 (60%)	5	22
	RGKO	5/5 (100%)	3	7
**mOS-2**	BALB/c	3/5 (60%)	1	10

# One hemangiosarcoma cell line (mHS-3) derived from RGKO-p53^−/−^ mice and two osteosarcoma cell lines (mOS-1 and mOS-2) derived from RGKO-p53^+/−^ mice were used. All mice were challenged with 10^6^ cells s.c.

* median weeks at sacrifice of tumor positive mice, when tumor volume reached 1.7 cm^3^.

**Table 2:** Metastatic ability of hemangiosarcoma and osteosarcoma cell lines in immunocompetent BALB/c and in immunodeficient RGKO mice

**Cell line**	**Host**	**Number of cells injected i.v.**	**Time to sacrifice(Median weeks after treatment)**	**Lung metastases**		
				**Incidence**	**Median**	**Range**
**mHS-3**	BALB/c	10^5^	59	2/4 (50%)	1	0–3
		10^6^	59	5/5 (100%)	6	1–>200
	RGKO	10^5^	59	1/3 (33%)	0	0–3
		10^6^	30	1/3 (33%)	0	0–3
**mOS-1**	BALB/c	10^5^	58	3/3 (100%)	3	2–9
		10^6^	17	3/3 (100%)	>200	>200
	RGKO	10^5^	31	3/3 (100%)	15	10–20
		10^6^	17	3/3 (100%)	>200	>200

### Expression of p53 and p53-related tumor suppressor genes

**Figure 8:**
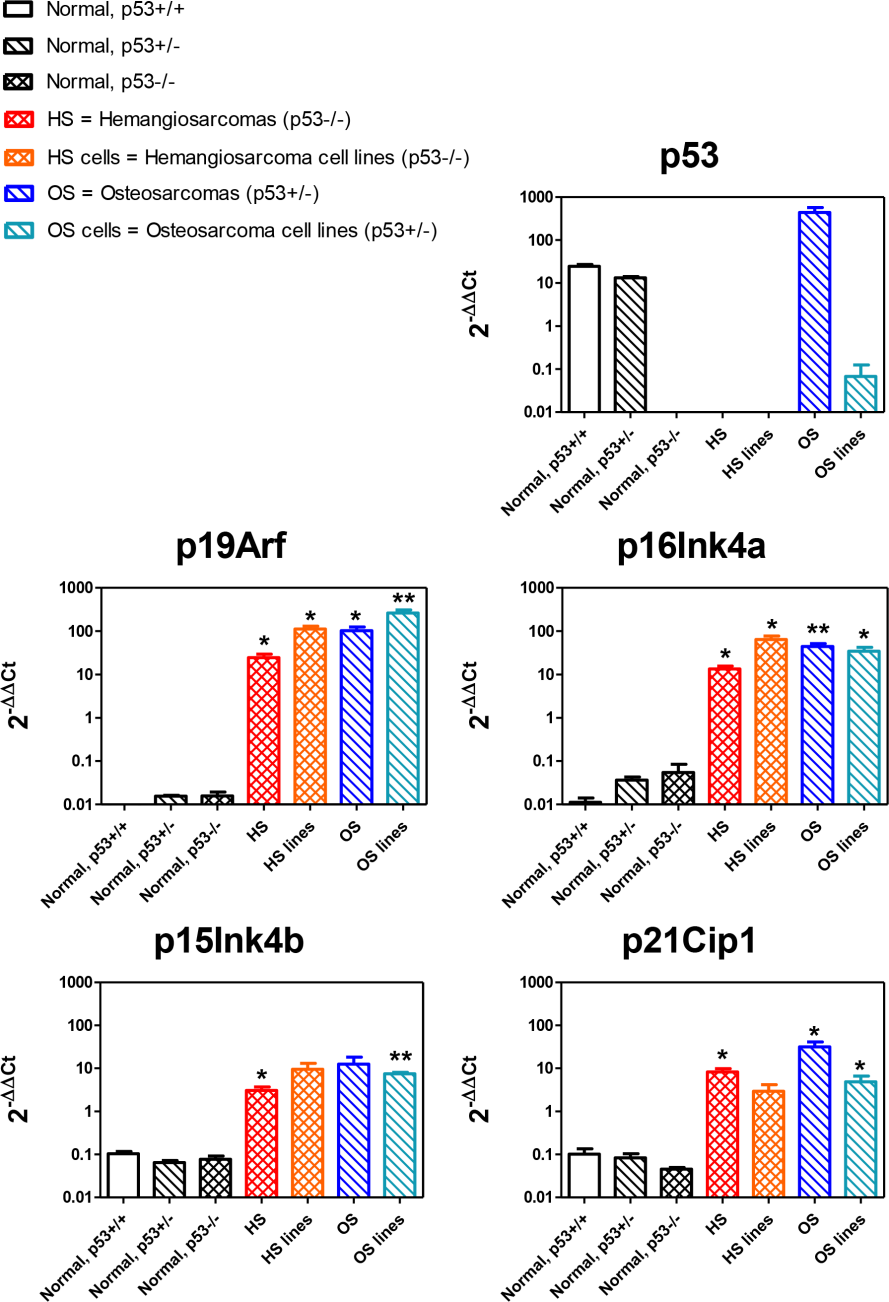
Expression of p53-related genes Each panel represents the expression of the indicated gene in hemangiosarcomas (from RGKO-p53^−/−^ mice) and osteosarcomas (from RGKO-p53^+/−^ mice) and in cell lines derived from these tumors, compared with gene expression in normal cells of mesenchymal origin (quadriceps muscle) of p53^+/+^, p53^+/−^ and p53^−/−^ carrying a different p53 status. Each bar in a panel represents the mean ± SEM gene expression level relative to the average of all samples (3–5 samples from different animals), normalized over GAPDH as endogenous reference gene. Statistical comparison between normal and tumor samples of p53^−/−^ and p53^+/−^ genotypes, *p < 0.05, **p < 0.01 Student's *t* test.

We analyzed the expression of p53 in normal tissues, tumors and cell lines obtained from mice carrying different p53 genotypes. As expected, p53 was absent in all samples from p53^−/−^ mice (Fig. 8). It is worth noting that osteosarcomas of p53^+/−^ mice retained p53 heterozygosity, which was instead lost in cell lines, a phenomenon known to occur also in human tumors [20].

We have recently shown that, in a different mouse model [21] sharing with the present ones the knockout of p53, various p53-related tumor suppressor genes were hyperexpressed [22], such as cyclin-dependent kinase inhibitor 1A (*CDKN1A/p21Cip1*), cyclin-dependent kinase inhibitors 2A (*CDKN2A/p19Arf*, *CDKN2A/p16Ink4a)* and cyclin-dependent kinase inhibitor 2B (*CDKN2B/p15Ink4b)*

[23–28]. Therefore we studied the expression of these genes in tumors and cell lines of RGKO-p53^−/−^ and RGKO-p53^+/−^ mice. We found that their expression was significantly enhanced in comparison to normal tissues of mesenchymal origin with different p53 status (p53^+/+^, ^+/−^, ^−/−^), thus confirming that the impairment of p53 causes an increase in the expression of related tumor suppressor genes (Fig. 8).

## DISCUSSION

Carcinogenesis in p53 knockout mice recapitulates the natural history of tumor onset in humans, starting with lymphoid malignancies, followed by sarcomas, then by carcinomas. However, in p53 knockout mice, the high prevalence and fast growth of lymphomas obscured all other tumor histotypes.

Thank to the fact that life without mature lymphocytes is possible, if the host is not exposed to deadly infections, we could strongly reduce the onset of lymphomas in p53 knockout mice by crossing them with alymphocytic Rag2^−/−^;Il2rg^−/−^ mice. This revealed a tumor landscape dominated by soft tissue sarcomas, in particular of vascular origin. It is interesting to note that the global incidence of carcinomas remained far below the incidence of sarcomas, thus suggesting that, as sarcomas were masked by lymphomas, now carcinomas are masked by sarcomas. If it were possible to genetically delete the mesenchymal cells that give rise to sarcomas, as we did with lymphoma precursors, all mice would probably develop carcinomas at a later age. It is worth mentioning that in p53 knockout mice a shift from lymphomas to sarcomas, and an increase in survival was obtained also by treatment with rapamycin the inhibitor of mTOR (mammalian Target Of Rapanycin) [29]. The activity of the mTOR pathway, usually inhibited by p53, seems to be increased in some tissues of p53^−/−^ mice with a tendency to elevated IGF-1 levels, but the effects of absence of p53 can be partially attenuated by rapamycin, leading to changes in tumor spectrum [29, 30].

The tumor spectrum of p53^+/−^ heterozygotes is completely different from that of p53^−/−^ homozygotes. Firstly, tumors arise much later in the life of heterozygotes. As these tumors usually show loss of heterozygosity at p53, the longer latency can be equaled to the time required for the loss of the wild-type p53 allele. Secondly, carcinomas dominate the tumor spectrum of heterozygotes. There could be several explanations for this fact, however the simplest one connects long latency with tumor histotype: loss of p53 heterozygosity occurs when mesenchymal cell populations are mostly quiescent, after the burst of proliferation required for growth to adult size, thus neoplastic transformation to sarcoma becomes relatively rare. For what concerns the tumor spectrum modifications introduced by the alymphocytic Rag2^−/−^;Il2rg^−/−^ genotype, as in p53^−/−^ mice, also in p53^+/−^ mice, this resulted in a reduction of lymphomas in favor of sarcomas.

The sizeable incidence of osteosarcomas in female RGKO-p53^+/−^ mice makes an interesting model system for the study of bone tumors. It is worth noting that epidemiological studies of human osteosarcoma show a typical bimodal age distribution of tumor onset, with a first peak in early adolescence and a second peak in adults over the age of 65. Osteosarcoma among the elderly is frequently related to Paget's disease or represents a second malignancy, in some instances caused by previous radiation therapy for the first tumor. The incidence of osteosarcoma generally is higher in males than in females, but in the case of osteosarcoma as a second malignancy some studies reported a slightly higher frequency in females [31]. The increased incidence of osteosarcoma in immunodeficient female p53^+/−^ mice could reflect the higher incidence of osteosarcoma as second tumor in women and, when compared to the lower incidence in female immunocompetent p53^+/−^ mice, could suggest a role for the immune response.

RGKO mice lack adaptive immune responses and innate responses are severely impaired, not only because they lack NK cells, but also because all the cytokines secreted by the various lymphoid subpopulations are absent. The fact that the profound immune deficiency of RGKO mice caused only minor variations in tumor latency of p53 knockout mice is a testament to the potency of the carcinogenic risk (or better, carcinogenic certainty) triggered by p53 alterations. In fact, the only detectable variations ascribable to the lack of immune surveillance were the slightly faster onset of tumors, in particular sarcomas, in RGKO-p53^+/−^ mice and the slower growth of tumor cells derived from the same tumors in immunocompetent *versus* immunodeficient hosts. The latter result could be attributed to the immunogenicity of tumors arising in the absence of immunoediting, *i.e*. the negative selection of immunogenic cell variants actuated by the immune system in immunocompetent mice [32].

RGKO-p53^−/−^ mice are a novel model system for the preclinical development of targeted therapies of human cancer with p53 alterations, which represent a prevalent molecular alteration of human tumors in need of novel targeted therapeutic approaches. A challenging set of new molecular targets can derive directly from the lack of p53, which frequently results in the derangement both of upstream p53 regulators and of downstream p53-regulated genes [22, 33–39]. In fact, various tumor suppressor genes, such as p21Cip1, p16Ink4a, p15Ink4b and p19Arf were up-regulated in sarcomas of RGKO-p53 mice, thus offering novel opportunities for the development of synthetic lethal therapeutic approaches.

In conclusion, we have shown that it is possible to modify the spectrum of tumors of p53 knockout mice which, in the absence of lymphocytes, develop a variety of solid tumors, in particular soft tissue sarcomas and osteosarcomas. The alymphocytic p53 knockout mice are a novel model system for studies of sarcoma development and for preclinical studies of targeted therapies.

## MATERIALS AND METHODS

### Ethics statement

All animal experiments were performed according European directive 2010/63/UE and Italian law (DL 26/2014). Experimental protocols were reviewed and approved by the Institutional Animal Care and Use Committee (“Comitato Etico Scientifico per la Sperimentazione Animale”) of the University of Bologna, and forwarded to the Italian Ministry of Health with letter 4783-X/10 (Responsible Researcher Prof. P. Nanni).

### Mice

BALB/c p53^+/−^ mice (BALB/cJ-Trp53tm1Tyj, originally obtained from The Jackson Laboratory, Bar Harbor, MI) have one p53 allele inactivated by the insertion of a neomycin resistance cassette in the region spanning exons 2 to 6 [5]. Rag2^−/−^;Il2rg^−/−^ breeders were kindly given by Drs. T. Nomura and M. Ito of the Central Institute for Experimental Animals (Kawasaki, Japan) [40, 41]. Mice were bred in our animal facilities under sterile conditions. BALB-p53^+/−^ mice were crossed with BALB Rag2^−/−^;Il2rg^−/−^ and the offspring were genotyped by PCR analysis of genomic DNA (see below for PCR conditions and primers). Selected progeny was used for subsequent crossings. Starting with the F2 generation, mice carrying genotypes of interest were weekly inspected for early signs of tumor onset (dyspnea, weight loss or suffering) in addition to meticulous palpation of limbs, mammary glands and peritoneum internal organs. Mice were sacrificed according to the criteria for humane endpoints approved by the Institutional Animal Care and Use Committee of the University of Bologna. At sacrifice, an accurate necropsy was performed. Brain, lung, heart, liver, adrenals, kidneys, spleen and lymph nodes, genitourinary tract, salivary glands, subcutis, muscles, mammary glands and limbs were macroscopically inspected and samples were collected of all visible tumors. Part of each tumor was fixed in 10% formalin, when necessary decalcified, and embedded in paraffin. The remaining tumor was divided into small samples and snap frozen. Two-three-micrometer paraffin sections were stained with hematoxylin and eosin and evaluated by an experienced pathologist.

### Cell lines, tumorigenic and metastatic potential

Osteosarcoma and hemangiosarcoma cell cultures were established from mechanically dissociated tumors. Hemangiosarcomas were cultured in DMEM (Dulbecco's Modified Eagle Medium) + 20% fetal bovine serum (FBS); osteosarcomas were cultured in IMDM (Iscove's Modified Dulbecco's Medium) + 10% FBS. All cells were maintained at 37°C in a 7% CO_2_ atmosphere. Tumor samples and cell lines were analysed by immunofluorescence and cytofluorometric analysis using the following anti-mouse antibodies: VEGF-R2 (clone Avas12α1), CD31 (clone MEC 13.3), CD146 (clone ME-9F1), CD45R/B220 (clone RA3-6B2), CD3 (clone 145-2C11), CD4 (clone GK1.5), CD8 (clone 53–6.7) all from Pharmingen BD Biosciences. To assess tumorigenicity and metastatic ability, cells were injected subcutaneuosly or intravenously in syngeneic immunocompetent or immunodeficient hosts. Mice were inspected weekly for tumor growth. Tumor volumes were calculated as π[√(*a*×*b*)]^3^/6 where *a* = maximal tumor diameter and *b* = major tumor diameter perpendicular to *a*. After sacrifice, an accurate necropsy was performed, then lungs were perfused with black India ink to outline metastases and fixed in modified Fekete's solution. Lung metastases were counted under a dissection microscope.

### Genotyping and real-time polymerase chain reaction

DNA for genotyping was isolated by digestion of tail tissue in a buffer containing proteinase K. To distinguish between the wild-type and mutant allele, two sets of primers were used for each gene: Wild-type *Il2rg* gene, forward CTG CTC AGA ATG CCT CCA ATT CC, reverse GAT CCA GAT TGC CAA GGT GAG TAG; mutated *IL2rg* gene, same forward primer, reverse CCT GCG TGC AAT CCA TCT TGT TCA AT (*neo* cassette) [42]. Wild-type *RAG2* gene, forward GGG AGG ACA CTC ACT TGC CAG TA, reverse AGT CAC TCT ACC TCT GAG GAC TGA; mutated *RAG2* gene, same reverse primer and forward CGG CCG GAG AAC CTG CGT GCA A (*neo* cassette) [43, 44]. Wild-Type *Trp53* gene, forward CCC GAG TAT CTG GAA GAC AG, reverse TAC TTG GCG GCT GGA TA; mutated *Trp53* gene same reverse primer and forward CTT GGG TGG AGA GGC TAT TC (*neo* cassette).

Gene expression was evaluated by quantitative real-time PCR using SYBR Green PCR Master Mix reagents (Applera, Milan, Italy) and analyzed by means of a Thermal Cycler Gene Amp 5700 Sequence Detection System (Applied Biosystem, Applera, Milano) using the following primer pairs: mouse *GAPDH* (forward, GCT CAC TGG CAT GGC CTT C; reverse, CGG TTC ATA CTA CTG TAG TTC TTC C); *TrP53* (forward, CCC GAG TAT CTG GAA GAC AG; reverse, TAC TTG GCG GCT GGA TA); *CDKN2A/p19Arf* (forward, GCT CTG GCT TTC GTG AAC ATG; reverse, CTA CTA CCC GTT GCA AGT GC); *CDKN2A/p16Ink4a* (forward, ACT CTT TCG GTC GTA CCC CG; reverse, CTA CTA CCC GTT GCA AGT GC); *CDKN2B/p15Ink4b* (forward, CCA ATC CAG GTC ATG ATG ATG G; reverse, GGT GCC TCG TCT TGG GTT); *CDKN1A/p21Cip1* (forward, GGA ACA TCT CAG GGC CGA A; reverse, TCT TTT GGG ACT TCA CGG GT).
